# An evidence-based knowledgebase of metastasis suppressors to identify key pathways relevant to cancer metastasis

**DOI:** 10.1038/srep15478

**Published:** 2015-10-21

**Authors:** Min Zhao, Zhe Li, Hong Qu

**Affiliations:** 1 School of Engineering, Faculty of Science, Health, Education and Engineering, University of the Sunshine Coast, Maroochydore DC, Queensland, 4558, Australia; 2 Center for Bioinformatics, State Key Laboratory of Protein and Plant Gene Research, College of Life Sciences, Peking University, Beijing 100871, P.R. China.

## Abstract

Metastasis suppressor genes (MS genes) are genes that play important roles in inhibiting the process of cancer metastasis without preventing growth of the primary tumor. Identification of these genes and understanding their functions are critical for investigation of cancer metastasis. Recent studies on cancer metastasis have identified many new susceptibility MS genes. However, the comprehensive illustration of diverse cellular processes regulated by metastasis suppressors during the metastasis cascade is lacking. Thus, the relationship between MS genes and cancer risk is still unclear. To unveil the cellular complexity of MS genes, we have constructed MSGene (http://MSGene.bioinfo-minzhao.org/), the first literature-based gene resource for exploring human MS genes. In total, we manually curated 194 experimentally verified MS genes and mapped to 1448 homologous genes from 17 model species. Follow-up functional analyses associated 194 human MS genes with epithelium/tissue morphogenesis and epithelia cell proliferation. In addition, pathway analysis highlights the prominent role of MS genes in activation of platelets and coagulation system in tumor metastatic cascade. Moreover, global mutation pattern of MS genes across multiple cancers may reveal common cancer metastasis mechanisms. All these results illustrate the importance of MSGene to our understanding on cell development and cancer metastasis.

Cancer metastasis is the ultimate step in cancer development, contributing to the majority of morbidity and mortality of cancer patients^1^,^2^. The interplay of tumor suppressor and oncogenes is one of the basic dogmas for cancer development. Similarly, there are co-existing genes to promote and suppress cancer metastasis. Metastasis suppressor genes (MS genes) generally refer to a class of cancer genes that inhibit the metastasis process without preventing primary tumour formation. NM23, the first identified MSG, mediates suppression of tumor metastatic process in melanoma cell lines^3^. In general, a series of cellular events are required to complete cancer metastasis. Any suppression along the metastatic cascade can interrupt metastasis^4^. Therefore, MS genes vary widely in their molecular functions and cellular locations. In terms of subcellular localization, MS genes may appear in extracellular, plasma membrane, cytosol, cytoskeleton, or intracellular organelles^5^.

As the invasive site are distinct from the site of primary tumor origin, the cellular micro-environments are also changed: e.g., O_2_ concentration, pH value, cytokines, growth factors, chemokines, etc.^6^. These differences may trigger multiple stress response events on both genetic and epigenetic level^7^. Starting from the outside of cell, extracellular matrix, a few MS genes are active on tissue invasion and matrix remodelling by controlling the matrix metalloproteases^4^. In general, integrin-mediated cell adhesions transduce the signals from extra cell to cytoskeleton. Therefore, numerous MS genes can suppress metastasis by interruption of the function of integrins. Along the integrated cellular signaling transduction to inner cell will further activate multiple stress-responding pathways, including c-jun-NH2-kinase (JNK), p38 signaling, and mitogen-activated protein kinase (MAPK) pathway. More interesting, numerous micro-RNA are identified as MS genes in cancers, which make the cellular signaling map more complex^8^.

Recently, an increased number of MS genes in various tumor types were characterized by using functional genomic techniques^9^,^10^,^11^,^12^,^13^. However, there are lacking the systematic study or comprehensive genetic resource to categorize known MS genes from abundant and diverse literature. Hence, the global functional view and the consistency for all the MS genes are not established across tumor types although steady accumulation of small-scale studies about MS genes. To address this challenge, we conducted a comprehensive evidence collection from PubMed abstracts. Our manual curation of the collected literature resulted a total of 194 human MS genes (161 protein-coding and 33 microRNA genes), and 1488 homologous genes from 17 model species. These curated MS genes are stored in the MSGene database (http://MSGene.bioinfo-minzhao.org/). These integrated MS genes with large-scale experimental evidence in various cancer types could provide a landscape of MS genes for genome-wide high-throughput screens. To keep pace with the growing demand for cancer genomics data integration, we provide quick access to MSGene with comprehensive functional annotations, such as COSMIC (somatic mutations from Catalogue of Somatic Mutations in Cancer)^14^, gene expressions from hundreds of tumors and normal samples from BioGPS (Gene Portal System)^15^ and methylation from DiseaseMeth database^16^. In addition, the online interface with user-friendly browser and query is also implemented for MSGene.

## Methods

### Extensive literature search for MS genes and literature curation

To provide a precise MS gene list with experimental evidence, we performed our literature search and curation as the following four steps: (i) We first performed an extensive literature query against PubMed (on Jan 20^th^, 2015) using complex expression: (“metastasis suppressor”[Title/Abstract] OR “metastasis suppressing”[Title/Abstract]) and (“cancer”[Title/Abstract] OR “tumor”[Title/Abstract] OR “carcinoma”[Title/Abstract]) AND ((“genome-wide association study” [Title/Abstract] OR “genome wide association study” [Title/Abstract]) OR (“gene”[Title/Abstract] AND (“association”[Title/Abstract] OR “microarray” [Title/Abstract] OR “expression” [Title/Abstract] OR “linkage” [Title/Abstract] OR “proteomics” [Title/Abstract] OR “genetic” [Title/Abstract] OR “metabolomics” [Title/Abstract] OR “copy number variation” [Title/Abstract] OR “hereditable” [Title/Abstract] OR “mouse model” [Title/Abstract] OR “animal model” [Title/Abstract] OR “microRNA” [Title/Abstract] OR “mutation” [Title/Abstract] OR “SNP” [Title/Abstract] OR “drug” [Title/Abstract] ))); (ii) As a result, 638 PubMed abstracts were obtained and grouped by the “Related Articles” function in Entrez system; (iii) We extracted text related to MS genes description from the grouped abstracts. Those text related to MS gene were manually read to extract the gene names and cancer type information with experimental evidence; (iv) The extracted candidate gene name and cancer type information were manually checked to classify the resulted genes and cross-check among different articles. After carefully checking manually, we consolidated 194 human MS genes (161 protein-coding and 33 microRNA genes) as core MS genes list from 550 PubMed abstracts. This core MS gene list will be regularly updated based on newly published literature.

### Biological functional annotation and database construction

To present the biological function involved and over-represented in our collected 194 MS genes, we retrieved comprehensive functional information from public resources (Table 1). The basic gene information and sequences are included and crosslinked to the NCBI Entrez gene^17^, UniProt^18^, Ensembl^19^ and Gene Ontology^20^. The mRNA expression profiling data from both normal and tumor tissues are imported from BioGPS^21^. To obtain comprehensive pathway-related information, we annotated the MS genes by using human protein atlas^22^, transporter substrate database^23^, BioCyc^24^, KEGG Pathway^25^, rate-limiting enzyme database^26^, PANTHER^27^, PID Curated^28^, pathway localization database^29^, PID Reactome^30^,^31^. The involved diseases were incorporated from GAD (Genetic Association Database)^32^, KEGG Disease^33^, Fundo^34^,^35^, NHGIR GWAS Catalog^36^, as well as OMIM^17^. In addition, the original MSG-related literature references in the NCBI PubMed database are hyperlinked to each gene. An automatic annotation pipeline was implemented to collect functional information from NCBI Gene/HomoloGene database^37^, Gene Ontology annotation, HPRD/BIND/BioGRID interaction annotation, KEGG LIGAND/BioCarta signaling event annotation^38^,^39^. The result shows that this automatic pipeline allows MS genes’ annotation to be easily updated when new information of relevant databases are available. Additionally, we will focus on constructing biological networks for human MS genes with emphasis on their regulatory transcription factors and protein-protein interactions.

### Gene set enrichment analysis

To assess the function of any interesting gene list, we conducted functional enrichment tests by using the online tool KOBAS^40^. KOBAS adopts a hypergeometric model to measure whether an input set of object pairs has a different frequency of annotation pairs than would occur randomly. Similar processes were used to identify enriched gene ontology. In these enrichment analyses, all the human protein-coding genes in KOBAS were used as background to calculate statistical significance. In addition, the Benjamini-Hochberg method was implemented in the KOBAS to further exclude false negative results. Finally, we collected those enriched functional terms with adjusted P-values less than 0.05.

### Gene ranking and cancer mutational landscape

We performed a gene prioritization using the ToppGene web server^41^ to help the user prioritize all 194 genes in MSGene. ToppGene requires two types of input. One is the training gene set, which contains genes already well-known MS genes. The other input is test gene set, which are the remaining interesting genes in our MSGene. To prioritize genes, ToppGene utilizes functional annotations in training dataset to calculate the similarity scores between test genes and genes in the training set. Multiple dimensional data is used to rank the input genes, including gene expression, regulatory information, functional annotations, sequence features, and literature mining data. It starts from extracting annotation features from the training genes that are well-known MS genes. To train the ranking model, we compiled a training gene list that included 11 genes (NME1, BRMS1, CD82, PEBP1, KISS1, NME2, CDH1, NDRG1, MTSS1, SERPINB5, CD44), which have at least 10 literature evidences. In the second stage, the ranking model was used to prioritize the remaining 183 genes using multiple annotation data. Finally, ToppGene combined all the rankings to a global ranking for all candidate MS genes using order statistics. The top 100 ranking MS genes, including 11 genes from the training set and 89 top ranked genes from ToppGene, are submitted to the cBio portal to present a mutational landscape across various cancer types^42^.

### Gene expression analysis in ovarian cancer

The ovarian cancer gene expression data with 489 high-grade serous samples was used to explore the gene expression change during cancer metastasis. The data set is generated from three gene expression microarray platforms (Affymetrix Exon 1.0 array, Agilent 244 K whole genome expression array, and Affymetrix HT-HG-U133A array)^43^. To present a unified gene expression, all the three datasets were normalized and calculated expression values for each sample and gene on each platform separately. After subtracting the mean value across samples for the same gene, the expression values were divided by the standard deviation across samples and the relative gene expression scores were obtained. Finally, the relative expression data from three platforms were integrated into a unified data set with 11,864 genes using a factor analysis model without batch effects^12^,^44^,^45^. The unified final gene expression data was downloaded from the TCGA website in a matrix format, in which one row for each gene and one column for each sample (https://tcga-data.nci.nih.gov/docs/publications/ov_2011/).

Based on the prepared gene expression matrix of ovarian cancer, we extracted the expression values of the MS genes in stage III and IV. In total, there are 142 MS genes overlapping to gene expression profiles from 381 stage III samples and 79 stage IV samples. We determined the expression changes of MS genes of the transition between stage III and IV by using the SAM package^46^.

## Results

### Web interface development and typical gene entries in MSGene

MSGene was constructed by using MySQL, the reliable open source relational database management system, to store all the MS genes, annotations, related data, and tools on a Linux server. The CGI Web-based interface using Perl is implemented in MSGene. Using the Perl CGI module and JavaScript technology, web pages for each gene in the database are generated.

**Figure 1:**
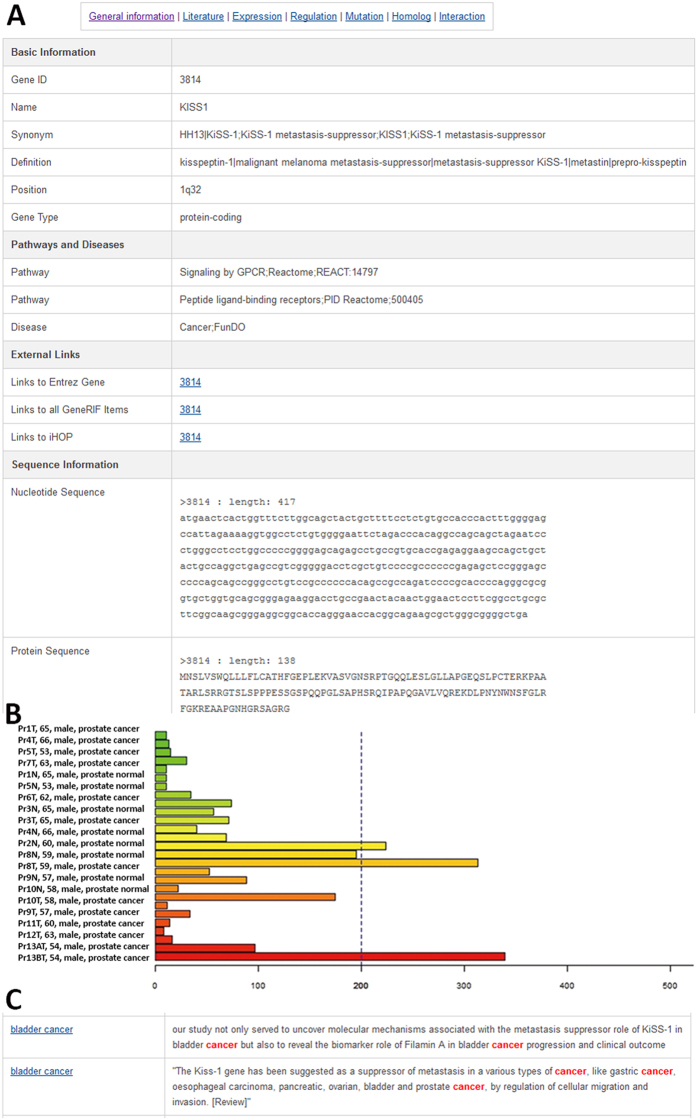
Gene information in the MSGene database. (**A**) Basic gene information in the MSGene database. (**B**) Gene expression in cancer samples. (**C**) A typical highlighted literature with supporting keywords.

As shown in Fig. 1, the annotations of a typical gene entry in MSGene can be categorized into seven types: “General information,” “Literature,” “Expression,” “Regulation,” “Mutation,” “Homolog,” and “Interaction.” By clicking on “General information” in each gene page, the user can access the gene name, involved pathways and diseases, nucleotide sequence, and protein sequence in a tabular view (Fig. 1A). In Expression label, gene expressions from normal tissues and cancer samples are provided as a bar graph with accompanying sample names (Fig. 1B). This bar graph is useful to acquire an overview of the expression specificity of each MS gene among different tissue types and cancer tissue/cell lines. Moreover, the extensive literature evidence associated with MS genes are also complied and highlighted with keywords related to MS gene or diseases in “Literature” view (Fig. 1C).

**Figure 2:**
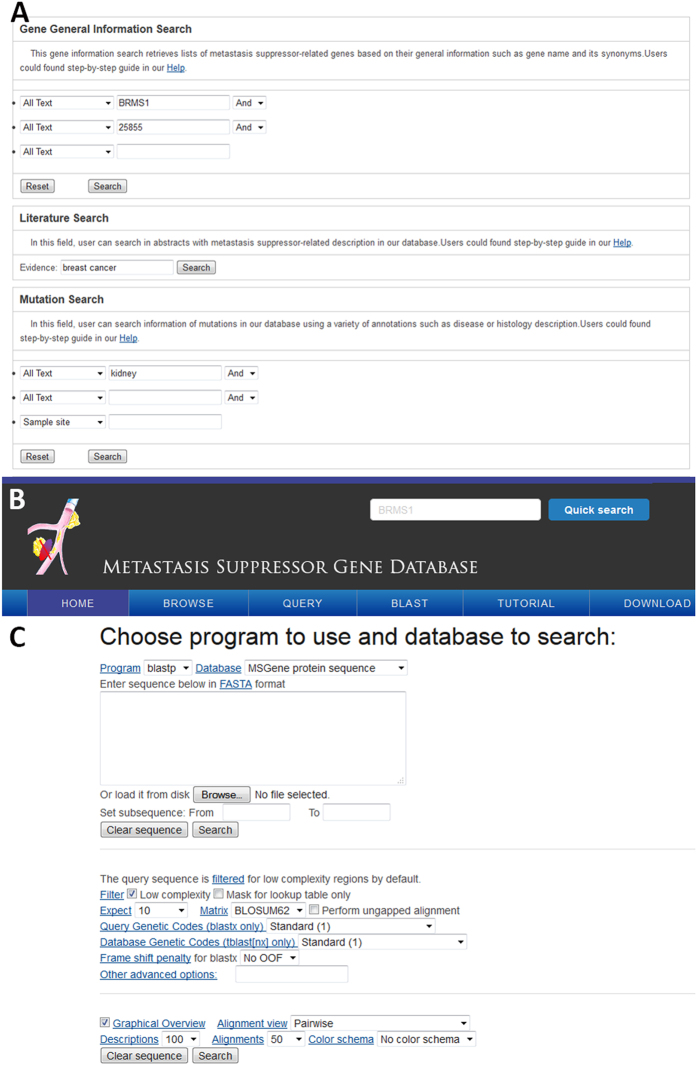
An interface for searching data from the MSGene database. (**A**) Keyword-based query interface. (**B**) Quick search button by gene name. (**C**) Sequence search via the BLAST interface.

**Figure 3:**
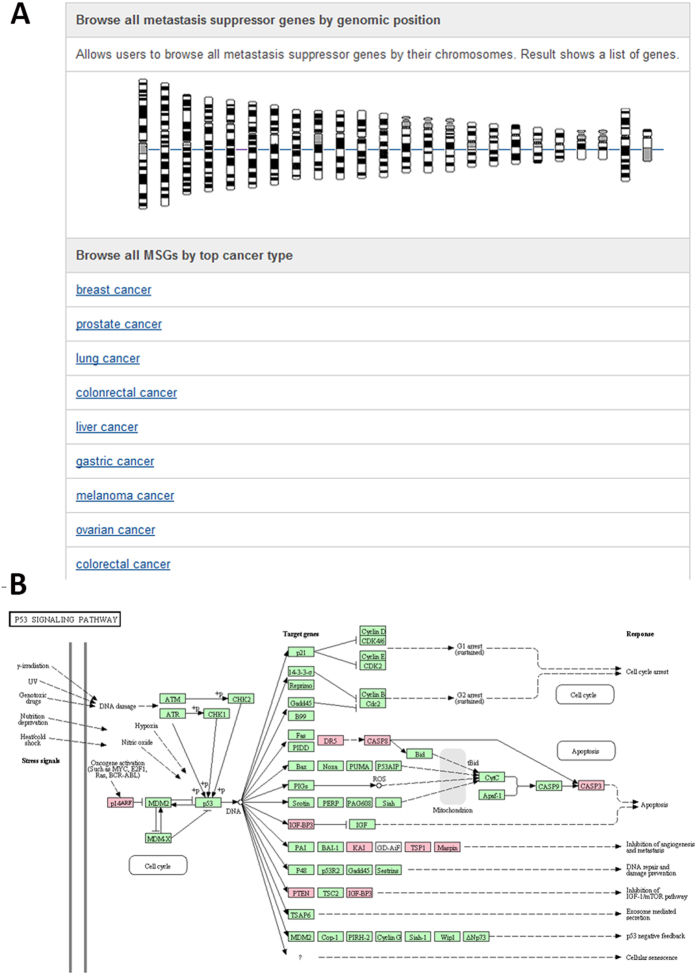
An interface for browsing data from the MSGene database. (**A**) Browsing MS genes by chromosome location and cancer type. (**B**) An example of browsing the data by pathway: KEGG p53 signaling pathway mapped with MS genes (pink color-marked) in the MSGene database. The pink color represents the genes which are included in our MSGene database. The green color represents the existence of the genes in human genome. The white nodes mean the genes are absent in human genome, but existing in our mammalian genome.

Our MSGene provides a user-friendly web interface to perform text query (Fig. 2A,B), or to run a sequence similarity search MSGene (Fig. 2C). In the text-based query page, six different powerful input forms are provided for the Entrez Gene ID, pathway and disease information, genomic location, literature evidence, and gene expression range in normal/cancer samples. Additionally, a quick text search for GeneID, gene symbol, and gene alias is on the top right of each page (Fig. 2B), which is convenient for a user to obtain any data in the database, especially literature-based annotations. Furthermore, users can browse the data in MSGene in a variety of ways, including significantly enriched pathway, related disease, reported linkage region, and chromosome number (Fig. 3). For each related KEGG pathway, the marked chart is provided to highlight all related MS genes. Finally, for any advanced study, MSGene provides all downloadable gene annotation and sequence information in a plain text format for all the collected 194 MS genes.

### Enriched biological pathways and subcellular localization for 194 MS genes

To better understand the function of these MS genes in our database, we performed pathway enrichment and disease association analyses on the 194 human MS genes to obtain general insights into their biological features using the KOBAS server. Over-represented pathways and significantly associated diseases were determined by using the hypergeometric test followed by the Benjamini-Hochberg multiple testing correction^40^. The enriched biological pathways and diseases with adjusted P-values less than 0.05 were collected. As shown in Table 2, the enriched pathways include cancer pathways (“MicroRNAs in cancer pathway,” “p53 signaling,” “Proteoglycans in cancer”). It is reported that proteoglycan content and distribution are markedly altered during cancer progression^47^. With specific structure in membrane, proteoglycan often interact with ligands and receptors that regulate cancer pathogenesis. Therefore proteoglycan, as well as glycosaminoglycans, often has profound roles in the tumor metastatic cascade by modulating key downstream signaling mediators such as epidermal growth factor receptor, insulin growth factor receptor, estrogen receptors, and Wnt members^48^. Interestingly, another three Reactome pathways are related to platelet, including “Platelet degranulation,” “Response to elevated platelet cytosolic Ca^2+^,” “Platelet activation, signaling and aggregation”. Accumulated evidences show that the activation of platelets and the coagulation system have a crucial role to support tumour metastasis^49^. With the protection of platelets, cancer cells may survive in the circulatory system from immune elimination. In addition, platelets can also help the establishment of secondary lesions at the endothelia cells. Other interesting pathways are related to apoptosis, including “Role of DCC in regulating apoptosis,” “TRAIL signaling,” “Extrinsic Pathway for Apoptosis,” and “Death receptor signalling”. It is a critical for MS genes to inhibit metastasis by controlling cell apoptosis^50^. In addition, the disease enrichment analysis associated two diseases (intracranial aneurysm and neoplasm metastasis) with MS genes. The intracranial aneurysm is a disorder with the weakness of cerebral artery or vein. In total, there are five MS genes related to intracranial aneurysm (CASP3, ENG, TIMP1, TIMP2, and TIMP3). It is worth noting that three genes are TIMP metallopeptidase inhibitor. To further assess the functional distribution of MS genes, we conducted enrichment tests on gene ontology terms. Using the complete human gene list as the background, the 194 MS genes were over-represented in 154 biological processes that were mainly clustering in epithelium/tissue morphogenesis and epithelia cell proliferation (Table S1). In summary, the level of complexity of cell surface and platelet signaling system involved in MS gene stems from the functions of components as fundamental roles in regulation of epithelium morphogenesis and proliferation.

We also collected all the subcellular localization information for the 194 MS Genes in human from the most recent subcellular localization analysis in human proteomics atlas (http://www.proteinatlas.org/)^22^. These information may help users to categorize the MS genes and have general ideas about where the MS genes are involved in metastasis. In total, there are 32, 29, and 13 MS genes mainly localized in nucleus, cytoplasm, and plasma membrane respectively. In addition, there are 8 MS genes localizing in vesicles, golgi apparatus, or endoplasmic reticulum.

### The common MS genes across multiple cancer types and also with the function of tumor suppressor genes

**Figure 4:**
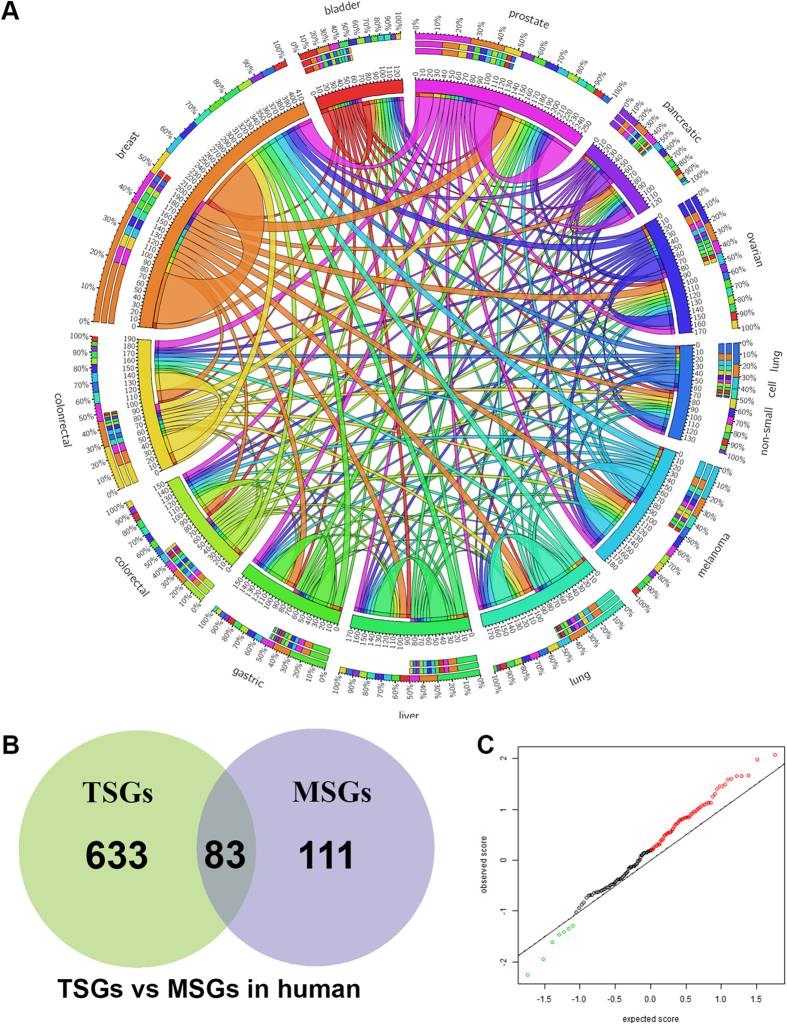
Global analysis of MS genes in multiple cancers. (**A**) The shared MS genes across 11 cancer types. The length of circularly arranged segments is proportional to the total MS genes in each cancer type. The ribbons connecting different segments represent the number of shared MS genes between cancer types. The three outer rings are stacked bar plots that represent relative contribution of other cancer types to the cancer type’s totals. (**B**) The overlapping relationship of tumor suppressors and MS genes in human. (**C**) The plot of differentially expressed MS genes between Stage III to Stage IV of TCGA ovarian cancer.

On the basis of information from the literature, we annotated all the genes in MSGene with cancer tissue information. We grouped all the MS genes into 58 cancer types. To explore the common mechanism of MS genes in different cancer types, we focused on the top 11 most abundant cancer types associated with >20 genes (Table S2). There is bias for the number of MS genes in those well- studied cancer types. Over half of MS genes (106) are characterized in breast cancer, colorectal cancer, and prostate cancer. Based on the common genes in the 11 cancer types, the overlapping relationships were plotted in Fig. 4A. It revealed that the multiple cancer types have common molecular mechanisms for metastasis suppressing. For instance, NME1 has been confirmed its metastasis suppressor role in 28 cancer types (Table S3). In total, we found 53 MS genes shared in at least 2 cancer types. The other common MS genes, including CD28, KISS1, NME2, BRMS1, shared in over 10 cancer types (Table S3).

Next, we test whether MS genes have any overlapping function with well-known tumor suppressors (TSGs). To this goal, we download 716 human TSGs from TSGene database^51^. We found 83 MS genes have been reported as TSG function (Fig. 4B). However, some well-studied MS genes such as NME1 and BRMS1 were also categorized as TSGs, which may need further experimental validation to confirm their dual roles as TSG and MSG. On the contrary, PTEN, another well-known TSG, was also reported to suppress metastasis in breast cancer^52^ and colorectal cancer^53^. For the remaining 111 MS genes, we run a functional enrichment analysis. Only one KEGG pathway and two gene ontology terms are significantly associated with the 111 MS genes. The pathway is MicroRNAs in cancer (Table S5, corrected P-value = 0.00014). The gene ontology terms are “extracellular matrix organization” (Table S4, corrected P-value = 0.02221) and “extracellular structure organization” (Table S4, corrected P-value = 0.02221). These results reveal that non-TSG MS genes have distinct extracellular localizations.

### The differential expression and mutation of MS genes during stage III to stage IV in ovarian cancer

By using public cancer transcriptome data, we further tested whether MS genes were differentially expressed during the metastasis. To this aim, gene expression data of 142 MS genes in ovarian cancer samples related to stage III to stage IV were extracted, which are created in the metastasis transition. In total, there are 70 genes having comparatively high expression by comparing the expression data in cancer samples in Stage III to Stage IV (Fig. 4C, Table S5). One of the biggest fold change is related to POSTN (fold change 25.14), which was reported to promote cell motility^54^. Another seven gens are detected with lower expression in stage transition. The most decreasing expression occurs for SMAD4. It has a negative fold change of −6.78 between samples from Stage III and Stage IV. SMAD4 has been reported to suppress invasion and metastasis by affecting expression of plasminogen activator inhibitor-1, E-cadherin and VEGF in ovarian cancer^55^. Those genes might be used as biomarkers for the ovarian cancer metastasis. Especially, the strong different expression of POSTN and SMAD4 from stage III to IV may mark the ovarian cancer metastasis process. The similar analysis can be applied to other cancer types when users have interests.

### Mutational landscape across multiple cancers based on the highly ranked MS genes

**Figure 5:**
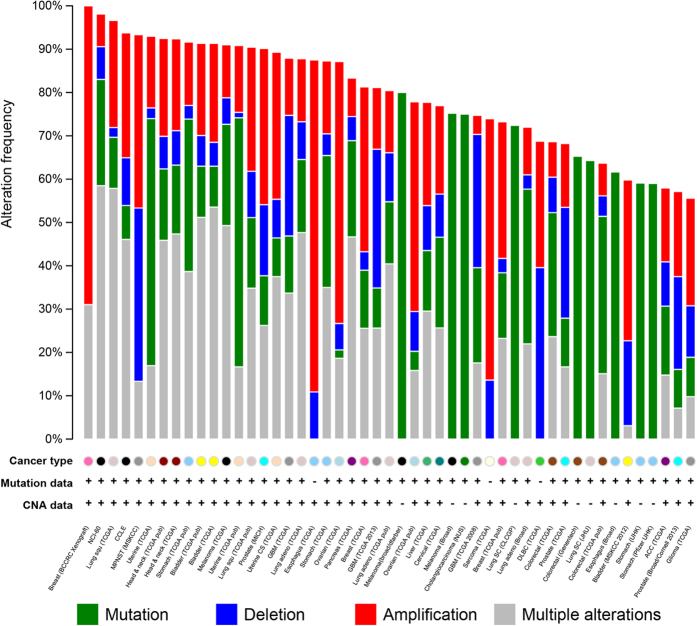
The mutational landscape for the top 100 MS genes in multiple cancers.

Although the 194 collected MS genes have literature evidence based on different experimental approaches such as abnormal gene expression, genetic study and animal models, the systematic examination of the importance of each MS gene has not yet been conducted. To this aim, we performed gene prioritization analysis for all the MS genes (Table S6). Besides 11 well-studied MS genes (NME1, BRMS1, CD82, PEBP1, KISS1, NME2, CDH1, NDRG1, MTSS1, SERPINB5, and CD44) in the training set (see Methods section), PTEN was top ranked MS gene in remaining 183 genes from the test set. A quick functional analysis on these top 100 MS genes (Table S7) show similar functional distribution with the total 194 MS genes (Table 2). The mutational frequency across multiple cancers may further confirm the importance of the gene ranking results. To this aim, the top 100 ranked MS genes were overlapped to cancer mutation data from cBio portal. As shown in Fig. 5, the top 100 ranking MS genes have overwhelming mutations (>50% mutation rate) in 50 cancer studies (Table S8). It is interesting that the 100 genes are over 90% mutated in cancer cell lines, including breast cancer patient xenografts, cancer cell line encyclopedia, and NCI-60 cell lines. Moreover, there are over 70% esophageal carcinoma patients with at least one amplification event on the top 100 MS genes, which may provide more clues about the metastasis of esophageal carcinoma.

Next, we explored the mutational frequency on a few well-known MS genes, including BRMS1, CD82, CDH1, KISS1 and NME1-3. As shown in Table S9 and Figure S1, BRMS1 has variations in 51 samples from 15 adult cancers (Acute myeloid leukaemia, Bladder, Breast, Cervical, Colorectal, Melanoma, Head & neck, Liver, Lung adenocarcinoma, Lung squamous cell carcinoma, Pancreas, Papillary renal cell carcinoma, Prostate, Stomach, Uterine cancer). Majority of these mutations are within the Sds3 domain, which is a conserved functional region for a set of transcription repressors. All the 35 mutations of CD82 locate within transmembrane domain, tetraspanin, which has roles in regulating platelet receptors. The top mutated MS gene is CDH1 (R-cadherin), which has been detected in 423 patients (Table S10). These hundreds mutations are distributed in all seven cadherin domains. However, the most famous MS genes, NME1, NME2 and NME3, only have 5, 1 and 9 mutations, respectively. This analysis revealed that CDH1 may have the prominent roles in the cancer metastasis in terms of their abundant mutational rate across multiple cancer types.

## Discussion

In this study, we constructed the first literature-based MS gene database, which currently contains 194 human genes curated from thousands of literature, importing high-throughput sequencing genetic and expression data. MSGene is the first attempt to establish a literature-based knowledgebase of MS gene with a user-friendly web interface, which provides users with a sophisticated text query, sequence search, browsing using functional analysis results, highlighted pathway maps and gene prioritization.

To test the MSGene, we applied an integrative systems-based approach to rank MS genes and compare with known tumor suppressors. The results support the overlapping roles of two type cancer suppressors. For example, one of most well-known tumor suppressor PTEN has been characterized as MSG. Our comparison may provide a clue of the common suppressing mechanisms between metastasis and cancer growth, which may elucidate common pathways for future drug development.

With the rapid increase in advanced gene and expression assays at high-throughput levels, the volume of data published related to cancer continues to expand. While the future of personalized medicine in cancer metastasis will include a systems biology approach, there is great opportunity at the population level as well. Complex genetic and genomic alterations may occur due to a wide variety of variants, including common variants, rare variants (mutations), and epigenetic phenomena. A systems biology approach will be necessary to integrate large volumes of data and determine the critical driver mutations that regulate activity as well as ultimately associate with cancer metastasis. At first glance, one might conclude that our initial test of the MSGene simply identified the pre-existing known pathways related to metastasis; however, deeper analysis showed substantially more information. The analytic approach made possible by the MSGene allows us to quickly identify the gaps between known MS genes and the available cancer genomics data, which will provide novel targets for future study. For example, our deep analysis on the most well-known MS genes revealed that NME1 only have 5 mutations from 3 cancer types. However, the literature have at least support its roles in 28 major cancer types. This huge gap may indicate NME1 may have other functional significance not caused mutations. These could be on the DNA methylation level, gene expression, or protein modification level. Thus, a free and open multimodal system that integrates DNA, RNA, microRNA, methylation, proteomics, metabolomics, and other resources related to NME1 may provide a new insight about its mechanisms for metastasis suppressing. In summary, interpretation of our study relies on the reliable candidate gene list for metastasis suppressing from the literature. With more large-scale genomic data, the integrative based approach will play more important roles to discover novel pathogenetic mechanisms.

MSGene can be used for multiple purposes, including: (i) obtaining literature-based and importance ranked gene lists for metastasis and relevant cancer types; (ii) reviewing comprehensive annotations, including genetic mutations, involved biological pathways, protein–protein interactions, transcription factor regulations, and post-translational modifications; and, (iii) a resource for high-throughput genetic and clinical tests to find MSG-related genetic variants. Overall, our curated MS gene list maps the genomic and cellular landscape for metastasis suppressing, providing a valuable resource for the cancer research community.

## Conclusions

MSGene is constructed as a free database and analysis server to enable users to rapidly search and retrieve summarized MS genes. The comprehensive functional enrichment analyses reveal that multiple signal events, which involved in epithelium/tissue morphogenesis and epithelia cell proliferation, are related to MS genes. Central questions should be focus on integration of various cancer genomics data to identify the common mechanisms for MS genes. The MSGene is freely available at http://msgene.bioinfo-minzhao.org/.

## Additional Information

**How to cite this article**: Zhao, M. *et al.* An evidence-based knowledgebase of metastasis suppressors to identify key pathways relevant to cancer metastasis. *Sci. Rep.* **5**, 15478; doi: 10.1038/srep15478 (2015).

## Supplementary Material

Figure S1

Table S1

Table S2

Table S3

Table S4

Table S5

Table S6

Table S7

Table S8

Table S9

Table S10

## Figures and Tables

**Table 1:** Annotation statistics for 194 human MS genes.

Data category	Related entries	Annotated MS genes	Content/sources
General information			
Human MS genes	194	194	Gene symbol, synonym, genomics position, gene type from Entrez gene database
Homologs	1448	161	Gene symbol and organism information
Literature	550	194	Curated literature evidence for MS genes
Function and regulation			
Pathway	1117	120	KEGG and HumanCyc database, etc.
Disease	1233	96	NHGRI GWAS catalog and GAD databases, etc.
Transcription factor regulation	2824	160	Regulatory information initiated by human transcription factors
Post-translational modification	703	102	Experimentally verified PTMs from dbPTM
Expression and methylation			
Cancer tissue	194	133	Expression in 184 cancer samples from BioGPS database
Normal tissue	290	151	Expression in 84 normal tissues from BioGPS database
Methylation	2313	151	Methylation in promoter regions from the DiseaseMeth database
Genomic variation and functional interaction			
Mutations	189,607	194	Somatic mutational records from COSMIC database
Signaling interactions	10,460	147	Protein-protein interactions from PathwayCommons

PTM: post-translational modification.

**Table 2:** The 17 enriched biological pathways and diseases for 194 MS genes.

Pathway/Disease Name	Database	*p*-value	Benjamini-Hochberg corrected *p*-value
Pathways			
MicroRNAs in cancer	KEGG	3.16E-17	1.94E-13
Platelet degranulation	Reactome	2.06E-05	0.004170147
Role of DCC in regulating apoptosis	Reactome	2.70E-05	0.004714507
Response to elevated platelet cytosolic Ca^2+^	Reactome	3.10E-05	0.00494768
inhibition of matrix metalloproteinases	BioCarta	4.11E-05	0.005866484
Extracellular matrix organization	Reactome	0.0001054	0.010318122
TRAIL signaling	Reactome	0.0001807	0.013194457
Dimerization of procaspase-8	Reactome	0.0002556	0.016503079
Regulation by c-FLIP	Reactome	0.0002556	0.016503079
Caspase-8 activation by cleavage	Reactome	0.0002556	0.016503079
Platelet activation, signaling and aggregation	Reactome	0.0002928	0.017411502
De novo pyrimidine deoxyribonucleotide biosynthesis	PANTHER	0.0003371	0.018942573
p53 signaling pathway	KEGG	0.0005346	0.025033317
p53 pathway	PANTHER	0.0006532	0.028619345
Proteoglycans in cancer	KEGG	0.0008741	0.035273524
Extrinsic pathway for apoptosis	Reactome	0.001352	0.048781856
Death receptor signalling	Reactome	0.001352	0.048781856
Diseases			
Intracranial aneurysm	FunDO	0.000185	0.01334844
Neoplasm metastasis	FunDO	0.0003232	0.018481418
