# Supplementary figures and images for: An evidence-based knowledgebase of metastasis suppressors to identify key pathways relevant to cancer metastasis
Source: Sci Rep. 2015 Oct 21;5:15478. doi: 10.1038/srep15478 (PMC4614344; doi:10.1038/srep15478)

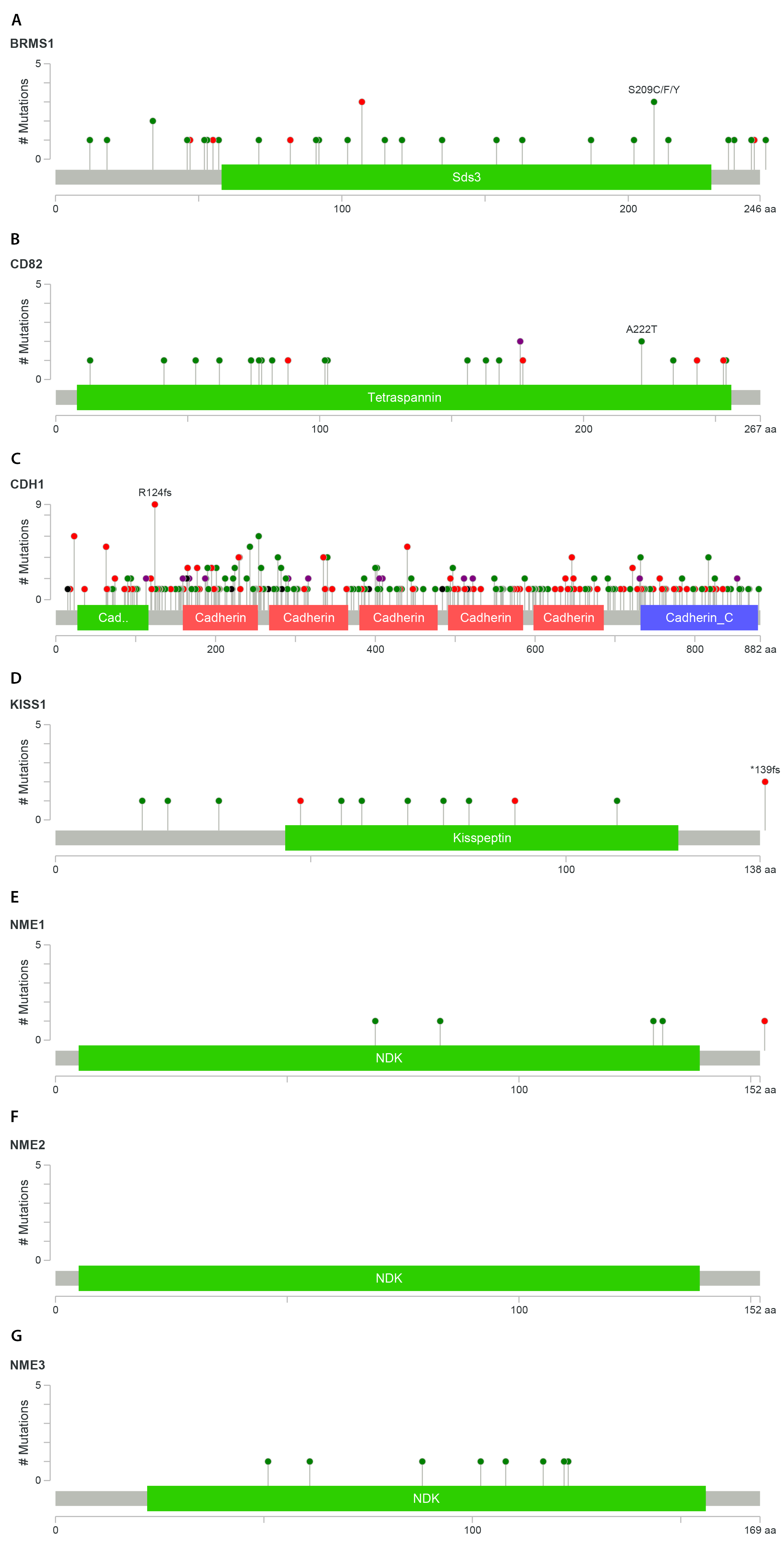

Supplement: Figure S1 [file srep15478-s1.gif]
